# Benefits of a bilingual web-based anatomy atlas for nursing students in learning anatomy

**DOI:** 10.1186/s12909-022-03405-8

**Published:** 2022-05-04

**Authors:** Meng-Lin Liao, Chi-Chuan Yeh, June-Horng Lue, Chung-Liang Chien, Shu-Hao Hsu, Ming-Fong Chang

**Affiliations:** 1 Department of Anatomy and Cell Biology, College of Medicine, National Taiwan University, 10051 Taipei, Taiwan; 2 Department of Surgery, National Taiwan University Hospital, College of Medicine, National Taiwan University, Taipei, 10051 Taiwan

**Keywords:** Anatomy, Bilingual, Nursing students, Self-study, Web-based learning

## Abstract

**Background:**

Registered nurses are required for high-quality healthcare. Thus, the anatomy course is essential regarding professional knowledge of the human body during the nursing training process. However, previous studies have indicated that anatomy teaching time and anatomy teachers were reduced and insufficient. Therefore, to improve the learning of practical anatomy in response to these difficulties, a bilingual National Taiwan University web-based anatomy atlas (NTU-WAA) was created as a cross-platform application and its feasibility was evaluated.

**Methods:**

The comparison of anatomy examination scores between nursing students of two cohorts (66 from the 2018–2019 cohort, whom was without NTU-WAA application; 54 from the 2019–2020 cohort, to whom NTU-WAA was offered) and the evaluation of questionnaires collected from nursing students of the 2019–2020 cohort and 4 anatomy teachers were carried out to define the feasibility of this strategy.

**Results:**

Results obtained by nursing students for the 2019–2020 cohort showed a significant increase in anatomy learning performance compared with that of the 2018–2019 cohort with reference to the laboratory midterm [2018–2019 cohort vs. 2019–2020 cohort, mean (standard deviation, SD): 77.20 (16.14) vs. 81.80 (12.03); *p* = 0.043], the laboratory final examination [59.68 (15.28) vs. 80.35 (13.74); *p* < 0.001] and the theory final examination [80.85 (10.10) vs. 84.33 (6.925); *p* = 0.017]. Moreover, results of the questionnaires indicated that the new bilingual cross-platform atlas was highly accepted by students and teachers.

**Conclusions:**

The NTU-WAA, a bilingual web-based atlas, was evaluated as a beneficial anatomy-learning tool that may enhance self-study of nursing students with consequent amelioration of their anatomy-related performance in both theoretical and laboratory examinations. This reflection suggests the future implementation of the bilingual web-based atlas on a large scale.

**Supplementary Information:**

The online version contains supplementary material available at 10.1186/s12909-022-03405-8.

## Background

Registered nurses play an integral and active role in the healthcare system. Registered nurses deliver the highest proportion of effective and curative patient care in healthcare organizations [1, 2]. However, the world is facing a critical shortage of registered nurses for the following reasons: aging of registered nurse workforce, growing elderly population, and a high turnover rate among younger nurses [3, 4].

An anatomy course encompassing theory and laboratory parts is the basic curriculum in a medical college. Students who plan to dedicate their career to medicine can gain the knowledge of the structures and functions of the human body through this course. The comprehension of anatomical knowledge could help learners when speaking of therapeutic concepts in the healthcare profession [5]. Nursing students can learn how to communicate with patients and other healthcare professionals in the context of diagnosis and treatment [6, 7]. Therefore, an anatomy course is required for safe clinical practice in healthcare professions.

Nursing students have pointed out that anatomical knowledge plays an important role in their practice [8]. However, a general perception reported by nursing students—including newly qualified nurses—has concerned inquietude and worries regarding biological sciences, including anatomy [9, 10]. Moreover, students usually declare low confidence in learning and applying anatomical and physiological information [11, 12]. According to previous studies, nursing students could benefit from exposure to human prosected specimens with reference to anatomical knowledge [5, 13, 14]; alternatively, anatomical laboratory experience may assist students in learning practical anatomy.

Because of inadequate curriculum time and shortage of anatomy teachers [15–18], several studies reported that the time for anatomy theory and laboratory classes was reduced [19–22]. For example, anatomy courses for nursing students at National Taiwan University (NTU) were only two laboratory classes (3 h per class) in which students had to identify many human structures via observing skeletons, plastic models, and prosected cadavers [23]. Therefore, to improve students’ learning of anatomy, the teachers had adopted some teaching tools in their courses, including plastic models, computer-generated images, social media, and other tools [24–28]. Previous studies also indicated that e-learning could be a useful educational tool to support anatomy learning [29–31]. Nevertheless, only a few online anatomy atlases designed for nursing students were available [30, 32].

English is thought to be a global language and medical professions usually learn medical knowledge and communicate in English. However, bilingual education is a growing global trend and is related with sociocultural factors [33–35]. Besides, some reports encouraged Asian universities to implement bilingual education strategy to avoid the English-only phenomenon [36, 37]. Moreover, information written in English language together with native language was reported to be an efficient learning approach [38]. Therefore, using native language with English in medical education could be incorporated into teaching/learning materials for better learning and good communication with the public.

Our previous study showed that the academic performance in anatomy laboratory of nursing students was not as good as that in anatomy lecture and the laboratory teaching time was limited [23]. Therefore, this study aimed to develop a bilingual web-based anatomy atlas combining traditional Chinese and English terminologies with the primary objective of helping nursing students to learn anatomy within limited teaching time. Furthermore, the evaluation of performance-related results obtained by nursing students was the second objective of this research.

## Materials and methods

### Creation and development of the web-based anatomy atlas

The workflow schema of the web-based anatomy atlas was performed as follows. First, the authors collected and organized a checklist of anatomical structures that are necessary for students of healthcare professions to identify according to lecture handouts used in the 2018 cohorts and suggestions from teachers that taught the corresponding chapters. The main anatomical systems have been mentioned in this atlas, including skeletal system, muscular system, cardiovascular system, respiratory system, digestive system, nervous system, urinary system, reproductive system, and special senses and endocrine system. Second, two experienced anatomy teachers used a digital camera (Sony RX100III, Zeiss lens 24–70 mm, F1.8–2.8) to acquire the photos of anatomical structures listed in the checklist at the first stage, selecting only good-quality and high-resolution files. The appropriate teaching photos were from human skeletal specimens, plastic models, separated skulls, sagittal section of skulls, and three dissected cadavers, preserved by the Graduate Institute of Anatomy and Cell Biology at NTU.

At the third stage, the same two teachers edited the aforementioned anatomical photos after a selection process and made detailed annotations by the means of Microsoft PowerPoint files. Eventually, the web-based anatomy atlas stage was realized at the fourth stage by an experienced anatomy teacher who could operate a Linux operation system and was familiar with HyperText Markup Language (HTML). The internet server was prepared in advance, and then authors adopted the HPE ProLiant ML30 Gen10 server (Hewlett Packard, Palo Alto, CA) with Community enterprise Operating System (CentOS 8) as a web-service server (The CentOS Project, https://www.centos.org/). A free website template (available for free at the following web address: https://www.themezy.com/free-website-templates) was selected to be used in this web-based anatomy atlas. Next, the PowerPoint files edited at the third stage were saved as JPEG files. These files were integrated into web-based anatomy atlas via Microsoft SharePoint Designer software. Finally, the NTU web-based anatomy atlas (NTU-WAA) was established and presented in the phase-shifting webpage structure (NTU-WAA, 2018). The website hyperlink is http://140.112.120.113/~mfc/NTU_WAA/

### Delivery of NTU-WAA

**Fig. 1:**
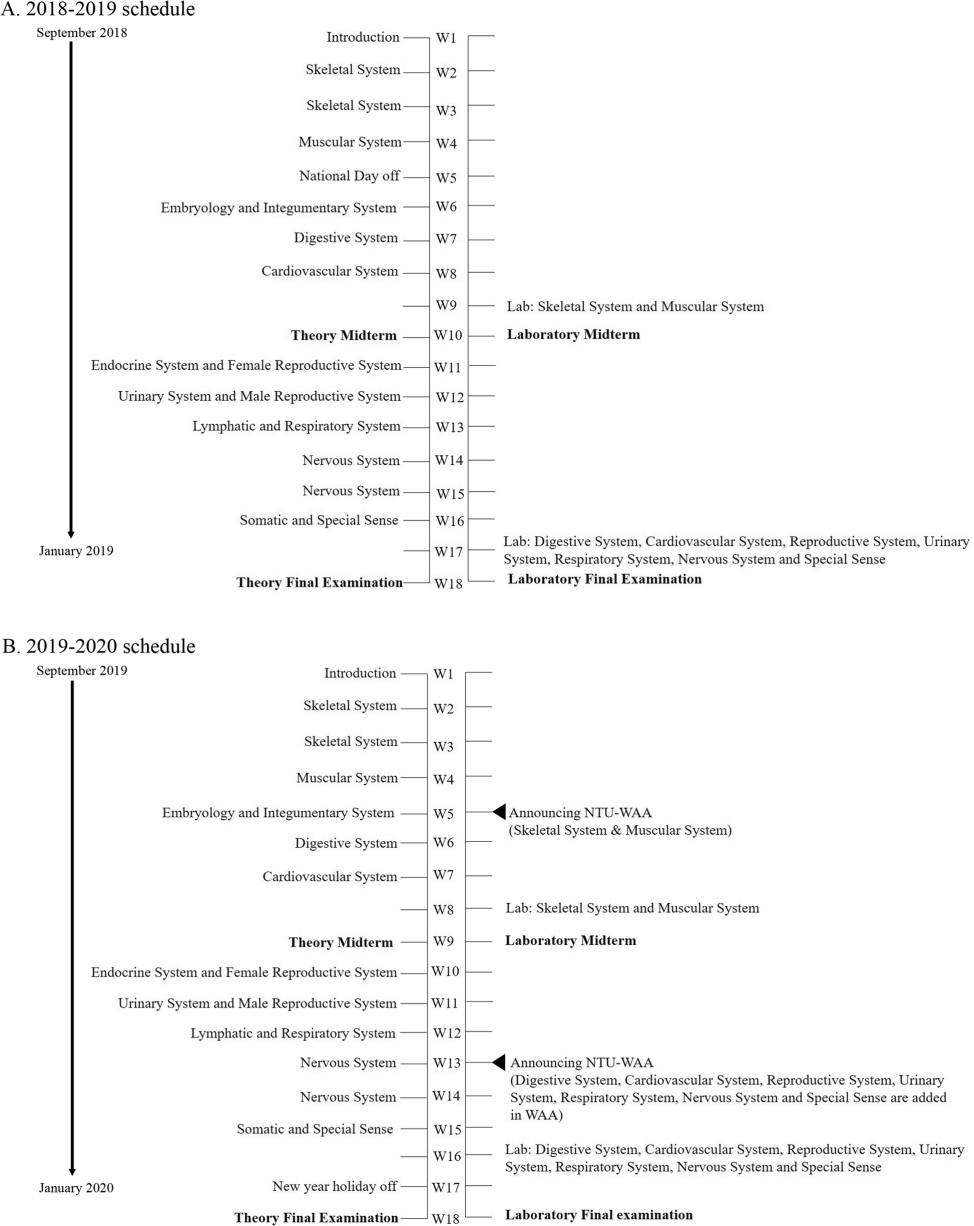
Schedule of NTU anatomy course for nursing students in the 2018–2019 cohort and the 2019–2020 cohort. There were 18 weeks in the first semester in 2018–2019 (A) and 2019–2020 (B) academic year of NTU, including 13-week lecture sessions, 2-week laboratory sessions, and 2-week examination time. The first part of NTU-WAA contained only skeletal and muscular systems and was released to students in the 5th week. By the 13th week, all nine chapters of NTU-WAA were completed and released to students

The curriculum schedules of the NTU anatomy course for nursing students for the semesters from September 2018 to January 2019 and from September 2019 to January 2020 were shown in Fig. 1. There were 13 lectures (39 hours) and two laboratory sessions (6 hours) in the anatomy course. Four examinations— theory midterm, laboratory midterm, theory final examination, and laboratory final examination—were arranged during the semester. The theory midterm covered the sessions before midterm, and the laboratory midterm included skeletal and muscular systems. The theory final examination contained the sessions after the midterm, and the laboratory final examination covered the following systems:

1. Digestive system
2. Cardiovascular system
3. Nervous system
4. Urinary system
5. Reproductive system
6. Respiratory system
7. Special senses and endocrine system

Based on the laboratory schedule of the 2019–2020 cohort, the skeletal and muscular systems of NTU-WAA were announced in the 5th week (W5) (3 weeks before the first laboratory class and 4 weeks before midterm examination), and the complete NTU-WAA with nine systems was released during the 13th week (W13) (3 weeks before the second laboratory class and 5 weeks before final examination).

### Analysis of the academic performance in the anatomy course

The academic performance was evaluated as per the scores of the anatomy course, which were collected from the Graduate Institute of Anatomy and Cell Biology, NTU. In total, 66 nursing students (29 students of Second-degree Bachelor of Science in Nursing [BSN] and 37 students of School of Nursing [SN]) from the 2018–2019 cohort and 54 nursing students (25 BSN and 29 SN students) from the 2019–2020 cohort signed the informed consent for data collection and agreed to participate in the study. The included participants were requested to conduct all anatomical examinations and no one was excluded.

There are two semesters, the first (September to January) and the second (February to June) semesters, in an academic year of the NTU, and the anatomy course for nursing students is in the first semester. In the 2018–2019 and 2019–2020 cohorts, the nursing students had the same schedule for the anatomy course with the same faculty members and similar examinations. The only difference was that nursing students of the 2019–2020 cohort had access to the NTU-WAA, while those of the 2018–2019 cohort did not. Regarding examinations, questions were designed by the anatomy teachers who taught the corresponding topics and the maximum score of each examination was 100 points. Every question in laboratory examinations was delivered through PowerPoint (time to answer: 1 minute). In this study, none of the photos in the NTU-WAA was used in aforementioned examinations. At the end of semester, the academic results of nursing students in the anatomy course were collected and analyzed using the Student’s t-test.

### Questionnaire about NTU-WAA for anatomy teachers and nursing students

The questionnaire about NTU-WAA for anatomy teachers (Supplementary Table 1) were delivered to faculties who did not take part in the creation and development of NTU-WAA during the 2019–2020 academic year, and four of them replied to the questionnaire.

The questionnaire about NTU-WAA for nursing students (Supplementary Table 2) was anonymously collected at the end of the first semester for the 2019–2020 cohorts and 54 nursing students wrote it.

### Evaluation of the difficulty of examination questions in two cohorts

Four anatomy teaching assistants who took the anatomy course before and did not assist in the preparation of anatomy examination answered the same theory and laboratory examination questions for the 2018–2019 and 2019–2020 cohorts in May 2020. Furthermore, to prevent the learning effect from answering examination sheets, testers simultaneously took the corresponding examinations of two cohorts with double answering time of nursing students. For example, testers answered two midterm theory sheets of 2018–2019 and 2019–2020 cohorts simultaneously. Their scores and mean scores of all examinations were analyzed via Pearson’s correlation coefficient and Student’s t-test, respectively.

## Results

### Establishing a bilingual web-based anatomy atlas in cross-platform

**Fig. 2:**
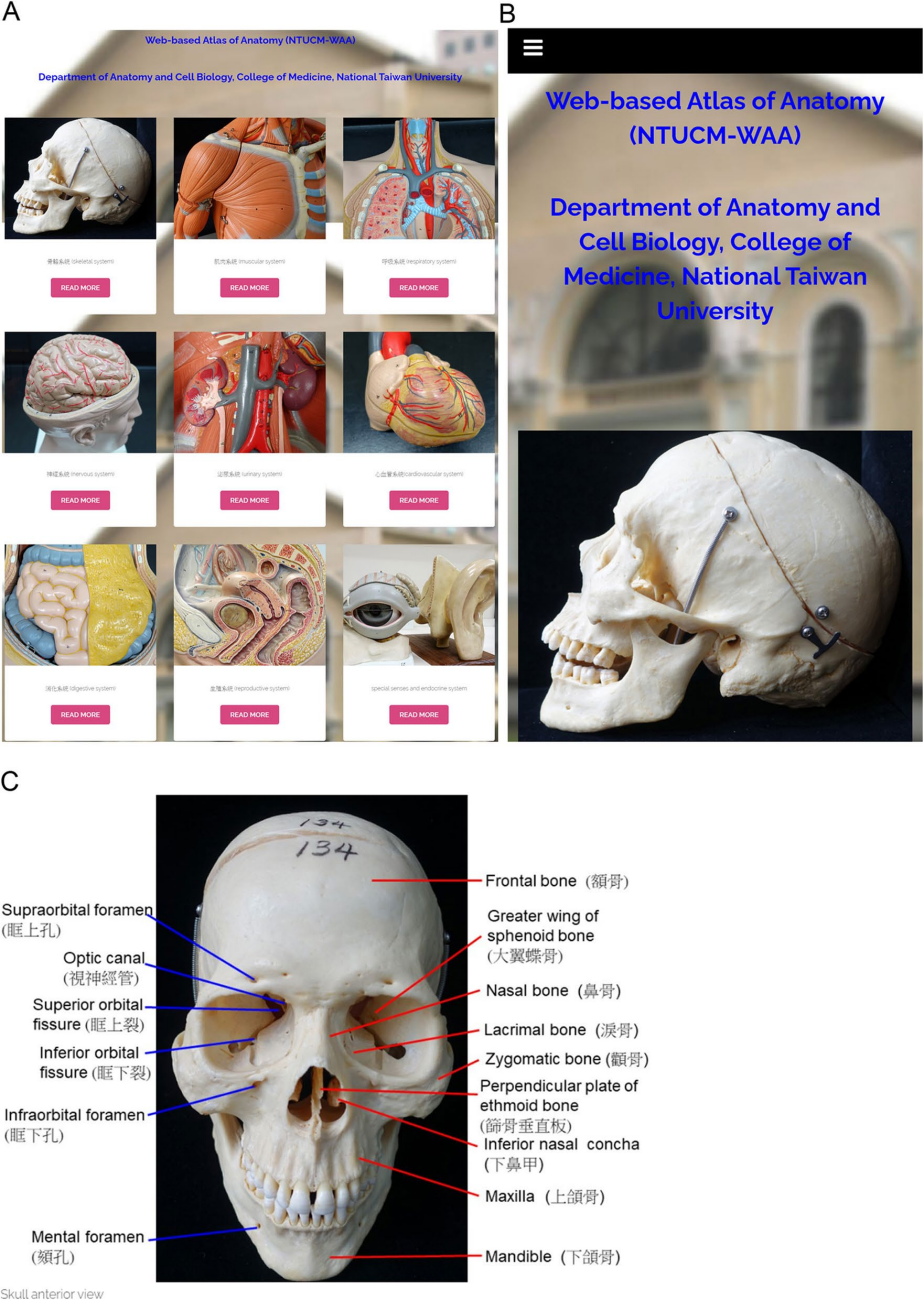
The main menu of bilingual NTU-WAA. The cross-platform software was designed with a cross-platform platform and could automatically shift between computers (**A**) and mobile devices (**B**), which was one of the characteristics of NTU-WAA. The second characteristic was that the anatomical terminology in the NTU-WAA was shown in English and traditional Chinese (**C**)

The authors created the cross-platform NTU-WAA software to assist nursing students in learning practical anatomy without time or place limitations. Because of the predictable high-usage rate of mobile devices by students, the user interface of NTU-WAA was designed to be able to automatically shift between desktop computers and mobile devices; alternatively, students could use NTU-WAA handily not only via computers (Fig. 2A) but also via mobile devices (Fig. 2B). Besides, a bilingual approach in medical education, which combined native language and English in written information, was implemented in NTU-WAA. Therefore, the labels concerning anatomical terminology in NTU-WAA were presented in English together with traditional Chinese (Fig. 2C). Hence, NTU-WAA has two basic features: cross-platform design and bilingual-terminology display.

### Evaluation of anatomical examination difficulty in two cohorts

**Fig. 3:**
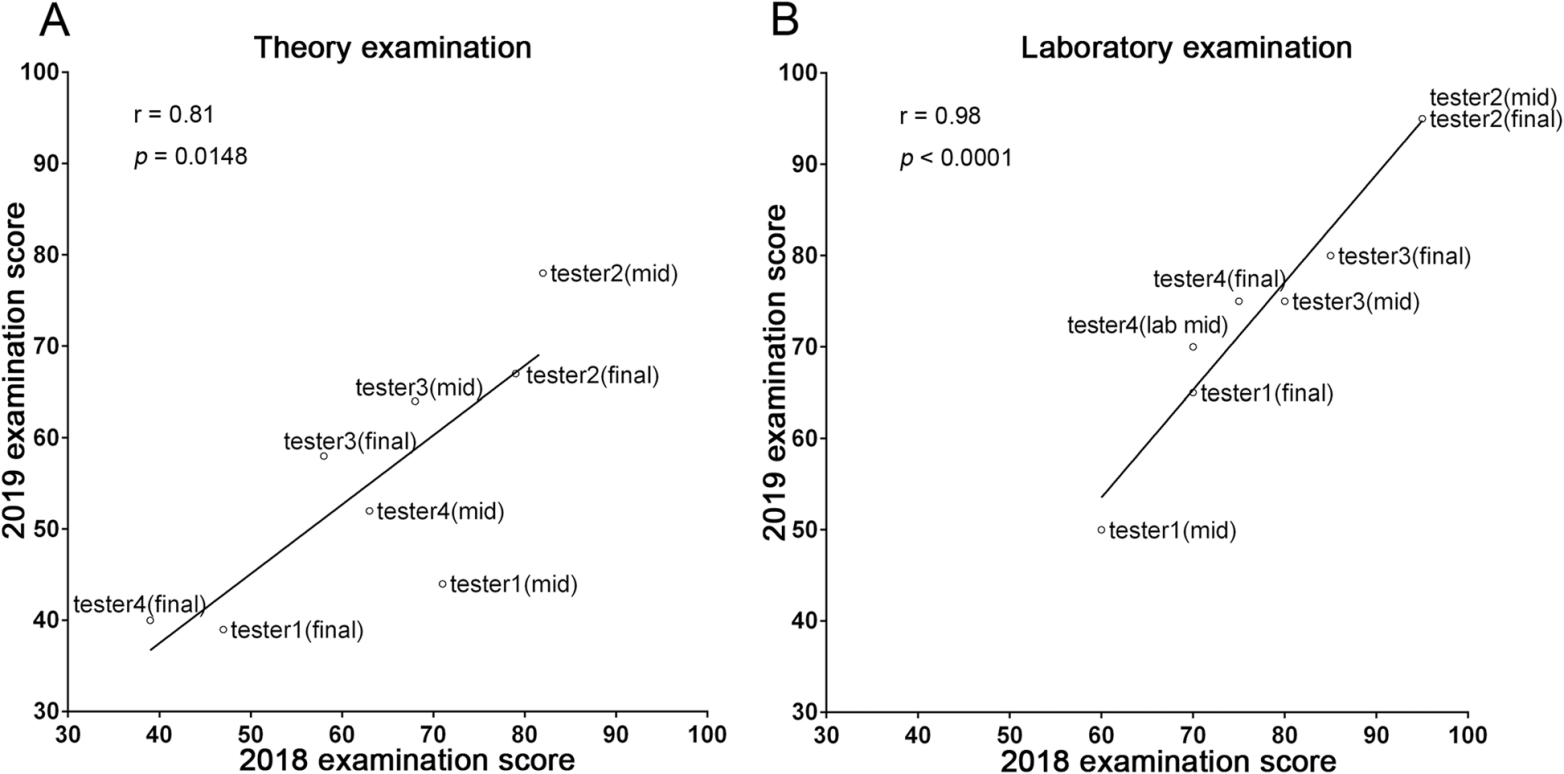
Evaluation of anatomical examination difficulty between the 2018–2019 and 2019–2020 cohorts. The difficulty of theory and laboratory examination of anatomy course between the 2018–2019 and 2019–2020 academic years was evaluated by four testers who were teaching assistants of anatomy course but blind to examination questions. Each score of testers’ midterms (mid) and final examinations (final) of two academic years was shown as a scatter plot. Pearson’s correlation coefficient (r) was used to measure the correlation of examination difficulty between the two academic years. Significant correlation was demonstrated if *p* < 0.05. The results of theory (**A**) and laboratory (**B**) examinations both showed strong correlation between the 2018–2019 and 2019–2020 cohorts.

The examination questions of corresponding topics of two cohorts were provided by the same teachers and were allocated with the same proportions of test scores, so the construct validity of each examination in the two cohorts was done by the experts. In order to show the fairness of comparison, the analysis of academic performances of nursing students between the two cohorts was carried out after an initial investigation concerning the difficulty of anatomical theory and laboratory examinations and related consistency between the two academic years. The scores of four testers showed that very strong correlation between the 2018–2019 and 2019–2020 academic years was observed both in theory (*r* = 0.81; *p* = 0.01; Fig. 3A) and laboratory examinations (*r* = 0.98; *p* < 0.0001; Fig. 3B). Moreover, there was no significant difference of mean scores between the two cohorts in the theory midterms, laboratory midterms, theory final examinations, and the laboratory final examinations (Table 1). The aforementioned results demonstrated that the difficulty of theory and laboratory examinations in the two academic years was consistent. Hence, the subsequent step was the evaluation of NTU-WAA benefits concerning academic learning through the comparison of the 2018–2019 and 2019–2020 cohorts.

**Table 1:** Mean scores of anatomical examinations obtained by four testers

Types of examinations		Mean scores (SD)		*P*-value
		2018–2019 cohort	2019–2020 cohort	
Midterm	Theory	71.00 (8.04)	59.50 (14.82)	0.12
	Laboratory	76.25 (14.93)	72.50 (18.48)	0.22
Final examination	Theory	55.75 (17.35)	51.00 (13.78)	0.23
	Laboratory	81.25 (11.09)	78.75 (12.50)	0.18

SD: Standard deviation

*P*-value < 0.05: Significant difference

### Comparison of the academic performance in the anatomy course between the 2018–2019 and 2019–2020 cohorts

**Fig. 4:**
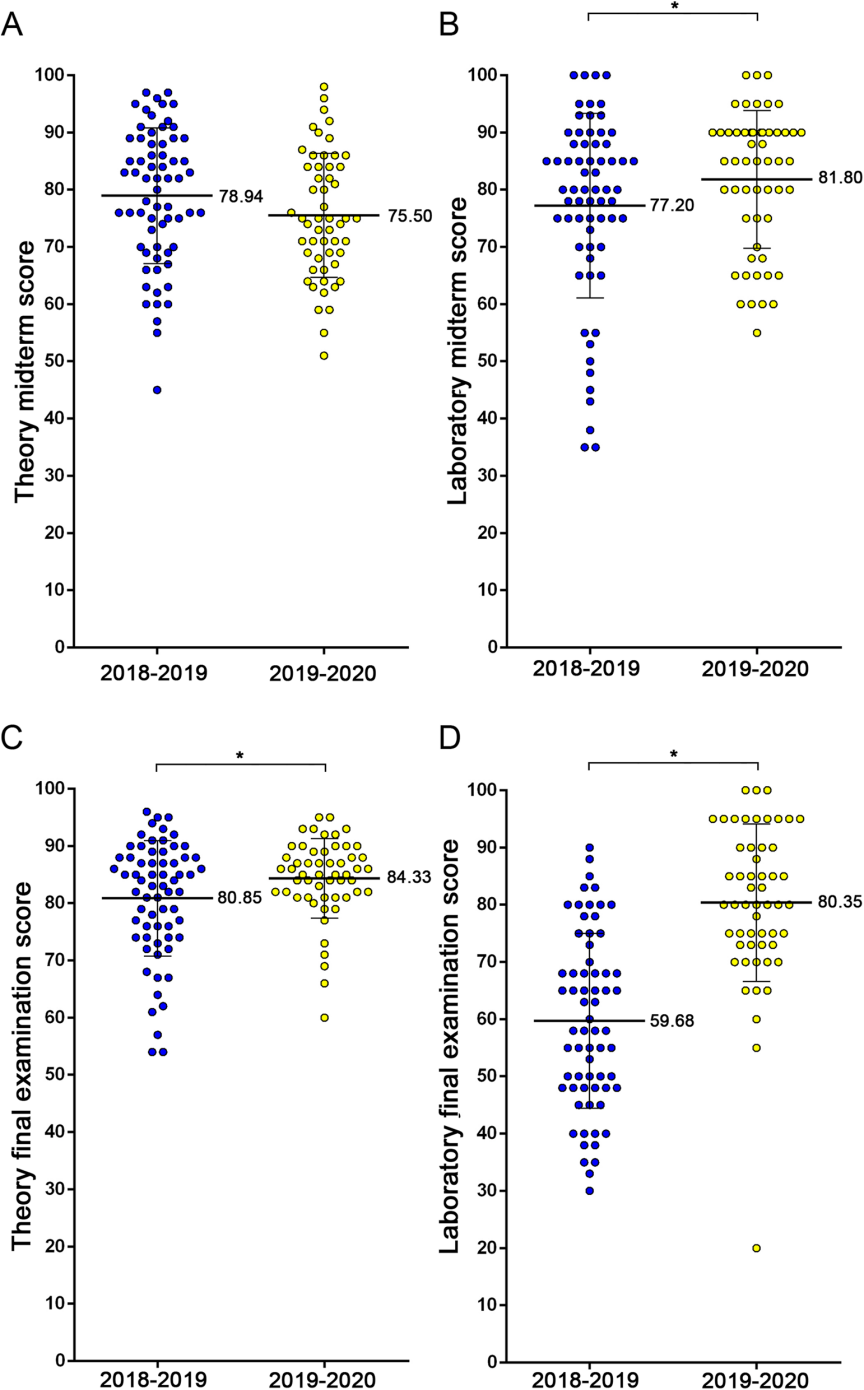
Comparison of the academic performance in anatomy course of nursing students of the 2018–2019 and 2019–2020 cohorts. The examination scores of nursing students in theory midterm (**A**), laboratory midterm (**B**), theory final examination (**C**) and laboratory final examination (**D**) were separately compared between 2018-2019 and 2019–2020 cohorts and were demonstrated by means (SD) of dot plots. The academic performance of nursing students of the 2019–2020 cohort were significantly increased in the laboratory midterm, the theory final examination and the laboratory final examination, whereas there was no difference in the theory midterm scores between the two cohorts. *, *p* < 0.05

To understand whether NTU-WAA could improve the anatomy learning of nursing students, the authors compared examination scores in the anatomy course of nursing students between the 2018–2019 and 2019–2020 cohorts (Fig. 4). Although there was no significant difference between two cohorts in the theory midterm scores (2018–2019 cohort vs. 2019–2020 cohort, mean (SD): 78.94 (11.83) vs. 75.50 (10.82); *p* = 0.1; Fig. 4A), the scores of nursing students in the 2019–2020 cohort were significantly higher than those in the 2018–2019 cohort regarding the laboratory midterm [77.20 (16.14) vs. 81.80 (12.03); *p* = 0.043; Fig. 4B], theory final examination [80.85 (10.10) vs. 84.33 (6.925); *p* = 0.017; Fig. 4C], and laboratory final examination [59.68 (15.28) vs. 80.35 (13.74); *p* < 0.001; Fig. 4D].

### Feedbacks by anatomy teachers and nursing students for NTU-WAA

To understand the perception of anatomy teachers and nursing students about NTU-WAA, questionnaires were collected from four teachers and 54 nursing students during the 2019–2020 academic year (Table 2). The results showed that anatomy teachers all agreed or strongly agreed for Questions 2–8. Besides, the results of collected questionnaires about the NTU-WAA from nursing students of 2019–2020 cohort showed that only 7.41% of the nursing students stayed neutral, whereas 14.81 and 77.78% of them agreed and strongly agreed, respectively, to include NTU-WAA in laboratory classes. Moreover, 26% of the nursing students used NTU-WAA for > 5 hours, 37% for 3–5 hours, 26% for 1–3 hours, and only 11% for 0.5–1 hour.

**Table 2:** Statistical analysis of feedbacks from anatomy teachers and nursing students for NTU-WAA

Questions for anatomy teachers	Percentage (%)(Number of anatomy teachers)					
	Strongly Agree	Agree	Neutral	Disagree	Strongly Disagree	
Q2. I agree to incorporate this platform in anatomy laboratory sections to assist students in learning anatomy.	75 (3)	25 (1)	0	0	0	
Q3. I agree that NTU web-based anatomyl atlas help students learn anatomy theoretical knowledge.	50 (2)	50 (2)	0	0	0	
Q4. I agree that NTU web-based anatomy atlas help students learn anatomy laboratory knowledge.	50 (2)	50 (2)	0	0	0	
Q5. I agree that NTU web-based anatomy atlas is helpful for teachers to teach theoretical knowledge.	50 (2)	50 (2)	0	0	0	
Q6. I agree that NTU web-based anatomy atlas is helpful for teachers to teach in laboratory classes.	75 (3)	25 (1)	0	0	0	
Q7. I agree that NTU web-based anatomy atlas is helpful for students to pass lecture examination.	50 (2)	50 (2)	0	0	0	
Q8. I agree that NTU web-based anatomy atlas is helpful for students to pass laboratory examination.	50 (2)	50 (2)	0	0	0	
**Questions for nursing students**	**Percentage (%)(Number of students)**					
Q1. I agree to add NTU web-based anatomy atlas in core-anatomy laboratory sections.	Strongly Agree	Agree	Neutral	Disagree	Strongly Disagree	
	77.78 (42)	14.81 (8)	7.41 (4)	0	0	
Q2. How long did you use the NTU web-based anatomy atlas after class in this semester?	Never used	< 30 minutes	30–60 minutes	1–3 hour(s)	3–5 hours	> 5 hours
	0	0	11 (6)	26 (14)	37 (20)	26 (14)
Q3. NTU web-based anatomy atlas is helpful for me in learning anatomy theoretical knowledge.	Very helpful	Helpful	Neutral	Slightly helpful	Helpless	
	62.96 (34)	29.63 (16)	5.56 (3)	1.85 (1)	0	
Q4. NTU web-based anatomy atlas is helpful for me in learning anatomy laboratory knowledge.	Very helpful	Helpful	Neutral	Slightly helpful	Helpless	
	68.52 (37)	25.93 (14)	5.56 (3)	0	0	

Total anatomy teacher number = 4

Total student number = 54

Furthermore, the questionnaire provided the information of what attitude the nursing students have toward NTU-WAA in learning practical anatomy with relation to theoretical and laboratory knowledge. The analysis data demonstrated that 92.59% of nursing students agreed that NTU-WAA was helpful (29.63%) or very helpful (62.96%) in understanding anatomy lecture, whereas 94.45% of them had positive attitude toward the helpfulness of NTU-WAA in laboratory sections (helpful: 25.93%; very helpful: 68.52%). Moreover, 5.56% of them stayed neutral about the NTU-WAA in learning anatomy theory and laboratory. Only one student (1.85%) perceived that NTU-WAA was only slightly helpful in learning theoretical knowledge.

From free-text feedbacks, anatomy teachers and many students provided several valuable suggestions and positive comments for the NTU-WAA (Table 3) that could make NTU-WAA better and more complete.

**Table 3:** Suggestions and comments for NTU-WAA from experienced anatomy teachers and nursing students

**Experienced anatomy teachers**	1. Could increase the interactive mode. 2. Could add more descriptions and annotations to assist students in reviewing key points. 3. Could add schematic photos of whole body with supine or prone position and label the relative position of the identified structure in the photos. 4. Could appropriately integrate photos of plastic models into NTU-WAA as supplements. 5. Could appropriately add cross sections in the NTU-WAA.
**Nursing students**	**Accessibility** 1. This platform is very good and helpful. Thanks for establishing it. I usually used it during my commuting time. 2. This platform is helpful not only before laboratory class, but also after laboratory class. Thanks for designing it with heart. I really appreciate it. 3. It is helpful for self-learning after class. 4. This platform provides great help for me to understand anatomical laboratory knowledge. It is a wonderful learning platform. 5. It is great to be able to use it to review at home. 6. I could see structures that I did not identify during the laboratory class (even clearer).
	**Suggestions for contents** 1. Some photos could be enlarged. 2. Photos of some structures were shown too close to be identified. 3. NTU-WAA is good for use. Could make some labels clearer. 4. Could show structures with more details. 5. Could add more photos of one organ or one region from different views to enhance identification. Overall, NTU-WAA is a great learning platform.
	**Future perspectives** 1. Hoping the NTU-WAA could be continuously opened for us. 2. Hoping to be able to use the NTU-WAA after the end of the class. 3. Could incorporate a self-assessment question database into the NTU-WAA for students to practice.

## Discussion

In this study, we established an online learning tool, the bilingual NTU-WAA, especially for nursing students whose native language is Chinese. Moreover, we demonstrated its feasibility via the improvement in academic performance of nursing students in anatomy course and positive feedbacks from both nursing students and anatomy teachers.

### Features of the NTU-WAA in this study

To increase the convenience and efficiency in learning anatomy for learners, the NTU-WAA was designed for cross-platform access, meaning users could gain access to the NTU-WAA via computers and mobile devices. Previous studies usually developed computer-assisted learning tools to support anatomy learning [39–41]; however, currently, mobile devices are indispensable for students for social and learning purposes [42]. Therefore, the automatic phase-shifting characteristic of NTU-WAA may increase the using rate as learners could flexibly learn anatomy with their available devices. According to the questionnaires, every nursing student from the 2019–2020 cohorts used the NTU-WAA (Table 2), and one student even gave feedback that he/she usually used the NTU-WAA while commuting (Table 3), suggesting that the NTU-WAA is convenient for users.

The other feature of NTU-WAA is the bilingual-terminology display (concomitant use of traditional Chinese and English languages). English is a prevalent language in the medical field worldwide and most healthcare experts communicate with each other in English [43, 44]. However, English terminology may be an obstacle for the relationship between patients and physicians or for communication among different healthcare professions, which could be improved by speaking native language [38]. Moreover, a previous study showed that physicians could improve medical knowledge when their native language is used instead of English [45], suggesting that students could also benefit from native-language-written terminology. Because English and native language play their own distingue role in medical education, a hybrid approach in written medical knowledge was developed and reported to facilitate learner’s understanding [38]. Therefore, regarding the anatomy course, the bilingual display of medical terminology could be taken into consideration in medical education.

### Benefits for learning anatomy lecture and laboratory

The results of comparison of the academic performance between two cohorts suggested that NTU-WAA may improve nursing students’ anatomy learning both in the laboratory identification and lecture knowledge. The NTU-WAA, containing the anatomical pictures of well-dissected cadavers and some corresponding plastic models, was designed to improve the laboratory learning of anatomy for nursing students. Previous studies have showed that prosection could be beneficial for learning anatomy [5, 13, 14] despite the fact that only a few academic performances were evaluated. Moreover, using an e-learning tool to guide in the laboratory class was reported to be able to improve the laboratory learning for students of chiropractic program [46]. In the current study, authors showed specifically that experience concerning NTU-WAA with photos of prosected cadavers could benefit nursing students in learning laboratory anatomy (Fig. 4B and D). Consequently, it was suggested that the software could be applied as a supplementary learning tool for students of other healthcare professions at institutes without anatomy rooms or when school-learning is prohibited. However, we do not know whether the improvement in laboratory performance could also increase the confidence of nursing students in applying anatomical knowledge in the clinical situation, and this issue could be followed in future investigations.

As there was no description about theoretical knowledge in the NTU-WAA, it was beyond our expectation that the performance of theory final examination in 2019–2020 cohort was also significantly increased (Fig. 4C). Japanese medical students were reported to have improved both their knowledge and practical performance with the help of computer-assisted instruction (CyberPatient abdominal anatomy) when compared with the only textbook group [47], suggesting that enhancing the practical learning through the assistance of computers may correspondingly facilitate the comprehension of theoretical knowledge. Besides, one study showed that the combination of cadaver dissection and computer-assisted learning tools could improve students’ retention of anatomical knowledge [48]. Furthermore, a previous study reported that students appreciated their ability to repeatedly review how to identify anatomical structures without time- or space-limitation with the help of an e-learning tool [46]. Similar feedback was received in this study (Table 3). Consequently, we deduced that improvement of the ability to review laboratory knowledge anytime and anywhere may contribute to the enhancement of the lecture performance.

The academic performance of theory midterm did not show difference between the 2018–2019 and 2019–2020 cohorts as that of final theory did. One possible reason for the different learning effects between theory midterm and final examination was that less corresponding lecture systems were included in NTU-WAA before midterm. Only skeletal and muscular systems were released on NTU-WAA before the midterm, but the lecture before midterm also covered cardiovascular, digestive, embryology and integumentary systems. In contrast, the NTU-WAA was well established with all systems covering the corresponding topics in the theory final examination. Therefore, the low number of covered systems in NTU-WAA before midterm may be one of the reasons for no improvement in midterm theory performance. To ascertain this result, we could release all units of the NTU-WAA at the beginning of the academic year in our next study.

Self-study may play a role in the aforementioned improved academic performance after the release of NTU-WAA. Students could use the NTU-WAA at will without help from instructors and use it depending on their own needs, their ability and their learning strategies. In this study, we provided the opportunity of self-study to nursing students via the release of NTU-WAA, which all students reported to use and 63% of them used for > 3 hours (Table 2). A self-study module for histology laboratory classes, which multiple audiovisual modalities and a virtual microscope platform were applied, was suggested to be helpful in recreating the experience of in-person laboratory classes and not to affect students’ examination performance when teaching hours were reduced [49]. Besides, a report showed that students increased their self-study time in anatomy via multimedia during COVID-19 pandemic may be helpful in understanding anatomic structures and enhancing dissection process [50]. Furthermore, independently self-study learning modules using three-dimensional constructs were also reported to be able to enhance students' performance and understanding of anatomy [51]. Thus, increasing the chances of self-study for students could be the target for anatomy instructors.

### Feedbacks about NTU-WAA by anatomy teachers and nursing students

Most comments about NTU-WAA from anatomy teachers and nursing students were positive and encouraging. From the questionnaire analysis, both teachers and nursing students agreed that the NTU-WAA was beneficial for learners in the theoretical knowledge and laboratory identification and could be continuously integrated into the anatomy courses. Moreover, some students even hope to be able to have access to the NTU-WAA after the anatomy course, suggesting that the NTU-WAA could be helpful for nursing students in learning anatomy course and probably in future clinical applications. Hence, a follow-up study about the benefit of NTU-WAA will be conducted with reference to nursing students commencing their clinical career.

This version of NTU-WAA did not have the interactive function between students and instructors, and an experienced anatomy teacher suggested that an interactive mode should be incorporated into NTU-WAA. Moreover, a student gave a suggestion that self-assessment question database could be integrated into the NTU-WAA. A recent paper reported that interactive lecture (including group discussion, tests, and answering questions) could enhance the learning and participation of nursing students [21]. Students missed the interaction with teachers and the time needed for prompt answers to questions in the classroom-learning during the distance-learning of COVID-19 pandemic [52], suggesting that interaction may play a role in students’ learning. Therefore, the NTU-WAA could incorporate an interactive function in the new version, such as message board and self-test assessment.

### Limitations of this study

This study had four limitations. First, the sample size was small because the NTU-WAA was announced during the 2019–2020 academic year, and thus data of only one academic year was collected. Therefore, the benefits of NTU-WAA in learning anatomy lecture and laboratory needs follow-up investigation. Second, the usage time of each nursing student was unknown because questionnaires were anonymous. Therefore, the individual improvement of academic performance could not be correlated to the actual usage time. Third, none of questionnaire questions explored the bilingual component of NTU-WAA and there was no web-based atlas without traditional Chinese terminology released to students, so the improved performance could not be directly related to the bilingual component. Further investigation on the bilingual effect could be conducted in the future. Finally, only nursing students were evaluated in this pilot study. For implementation on a large scale, the advantages of NTU-WAA in learning anatomy could be investigated among students of other majors, such as pharmacy, physical therapy, and occupational therapy.

## Conclusions

The bilingual web-based anatomy atlas, NTU-WAA, which combined traditional Chinese and English terminologies, was preliminarily established for nursing students in this study. We demonstrated that the NTU-WAA was a practical and helpful learning tool for nursing students in learning both anatomy theoretical and laboratory knowledge. This software could also be a useful teaching tool for anatomy teachers. In the future, the NTU-WAA will be continuously refined by adding more detailed and different anatomical photos, annotations, theoretical descriptions, and interactive functions to further enhance the anatomy learning of healthcare professions.

## Supplementary Information

**Additional file 1.**

**Additional file 2.**

## Data Availability

The data that support the findings of this study are available from the corresponding author upon reasonable request.

## Abbreviations

BSN: Second-degree Bachelor of Science in Nursing
NTU: National Taiwan University
NTU-WAA: National Taiwan University web-based anatomyl atlas
SD: Standard Deviation
SN: School of Nursing
