# Supplementary material for: Benefits of a bilingual web-based anatomy atlas for nursing students in learning anatomy
Source: BMC Med Educ. 2022 May 4;22:341. doi: 10.1186/s12909-022-03405-8 (PMC9064542; doi:10.1186/s12909-022-03405-8)
Supplement: Supplementary file 1 — Additional file 1. [file 12909_2022_3405_MOESM1_ESM.doc]

Supplementary table 1. Questionnaire for experienced anatomy teachers

| 1. I know the platform of NTU web-based anatomy atlas. □ Yes □ No |
| --- |
| 1. I agree to incorporate this platform in anatomy laboratory sections to assist students in learning anatomy.   □ Strongly Agree □ Agree □ Neutral □ Disagree □ Strongly Disagree |
| 1. I agree that NTU web-based anatomy atlas help students learn anatomy theoretical knowledge.   □ Strongly Agree □ Agree □ Neutral □ Disagree □ Strongly Disagree |
| 1. I agree that NTU web-based anatomy atlas help students learn anatomy laboratory knowledge.   □ Strongly Agree □ Agree □ Neutral □ Disagree □ Strongly Disagree |
| 1. I agree that NTU web-based anatomy atlas is helpful for teachers to teach theoretical knowledge.   □ Strongly Agree □ Agree □ Neutral □ Disagree □ Strongly Disagree |
| 1. I agree that NTU web-based anatomy atlas is helpful for teachers to teach in laboratory classes.   □ Strongly Agree □ Agree □ Neutral □ Disagree □ Strongly Disagree |
| 1. I agree that NTU web-based anatomy atlas is helpful for students to pass lecture examination.   □ Strongly Agree □ Agree □ Neutral □ Disagree □ Strongly Disagree |
| 1. I agree that NTU web-based anatomy atlas is helpful for students to pass laboratory examination.   □ Strongly Agree □ Agree □ Neutral □ Disagree □ Strongly Disagree |
| 1. Experience of teaching anatomy: ____ year(s); Age:_______; Gender: □ Male □ Female |
| 1. Do you have any suggestions or comments for NTU web-based anatomy atlas? |

A 5-point Likert Scale has been considered for Questions 2–8, from 5 meaning “Strongly Agree” to 1 meaning “Strongly Disagree.”
