# Supplementary material for: Benefits of a bilingual web-based anatomy atlas for nursing students in learning anatomy
Source: BMC Med Educ. 2022 May 4;22:341. doi: 10.1186/s12909-022-03405-8 (PMC9064542; doi:10.1186/s12909-022-03405-8)
Supplement: Supplementary file 2 — Additional file 2. [file 12909_2022_3405_MOESM2_ESM.doc]

Supplementary table 2. Questionnaire for nursing students

| 1. I agree to add NTU web-based anatomy atlas in core-anatomy laboratory sections.  □ Strongly Agree □ Agree □ Neutral □ Disagree □ Strongly Disagree |
| --- |
| 2. How long did you use the NTU web-based anatomy atlas after class in this semester?  □ Never used □ < 30 minutes □ 30–60 minutes □ 1–3 hour(s)  □ 3–5 hours □ > 5 hours |
| 3. NTU web-based anatomy atlas is helpful for me in learning anatomy theoretical knowledge.  □ Very helpful(5-point) □ Helpful (4-point) □ Neutral (3-point)  □ Slightly helpful (2-point) □ Helpless (1-point) |
| 4. NTU web-based anatomy atlas is helpful for me in learning anatomy laboratory knowledge.  □ Very helpful(5-point) □ Helpful (4-point) □ Neutral (3-point)  □ Slightly helpful (2-point) □ Helpless (1-point) |
| 5. Do you have any suggestions or comments for NTU web-based anatomy atlas? |
